# Marmosets contain multitudes

**DOI:** 10.7554/eLife.97866

**Published:** 2024-04-25

**Authors:** Kenneth Chiou, Noah Snyder-Mackler

**Affiliations:** 1 Center for Evolution and Medicine, School of Life Sciences, Arizona State University Tempe United States

**Keywords:** marmosets, genetic chimerism, single-cell RNA sequencing, sibling chimerism, microglia, Other

## Abstract

Single-cell RNA sequencing reveals the extent to which marmosets carry genetically distinct cells from their siblings.

**Related research article** del Rosario RCH, Krienen FM, Zhang Q, Goldman M, Mello C, Lutservitz A, Ichihara K, Wysoker A, Nemesh J, Feng G, McCarroll SA. 2024. Sibling chimerism among microglia in marmosets. *eLife* **13**:RP93640. doi: 10.7554/eLife.93640.

Marmosets almost always produce non-identical twins which, unusually, share much more than just a womb. The same circulatory system connects the siblings during pregnancy, allowing two genetically distinct individuals to freely exchange the hematopoietic stem cells that give rise to all blood and immune cells (Gengozian et al., 1969; Wislocki, 1939; Hill, 1932). As a result, a drop of blood from this tiny South American primate reveals a mixture of cells originating from each twin (Benirschike and Brownhill, 1962). This is known as sibling chimerism – from Chimera, the monster from Greek mythology that is a hybrid of a lion, a goat and other creatures.

This quirk of nature has been known for decades (Benirschke et al., 1962). Yet, while studies have detected genetic information from the other twin in certain organs, it has remained difficult to precisely test whether sibling chimerism is limited to blood-related cells or extends to other cell types (Sweeney et al., 2012; Ross et al., 2007). Answering this question would help to uncover how cellular exchange takes place between marmoset twins, while also allowing researchers to investigate how genetic variations affect cell function in vivo. However, this requires technology that has only become recently available, and which makes it possible to isolate and sequence the genetic material of individual cells in a single tissue. If different genomes are identified in a heart sample, for example, these newer approaches can now distinguish whether they originate from the muscle cells of the heart, the blood cells pumped through it, or the resident immune cells that protect it. Now, in eLife, Ricardo del Rosario, Steven McCarroll and colleagues at Harvard Medical School, the Broad Institute and MIT report having addressed this knowledge gap by applying single-cell sequencing to various marmoset organs (del Rosario et al., 2024).

Rather than focusing on DNA, the team took advantage of the thousands of genetic variants transcribed into RNAs and opted instead for single-nucleus RNA-sequencing. Being able to capture all the genes being transcribed in an individual cell did double duty. del Rosario et al. could identify which tissues contained cells from the other twin; and they could also examine the impact of these genetic differences on gene expression patterns and cell function.

To investigate the first question, the team tested for sibling chimerism in the blood, liver, kidney and brain – all tissues which contain varying amounts of cell types deriving from hematopoietic stem cells. This confirmed that chimerism is prevalent and widespread in marmosets, while also allowing del Rosario et al. to precisely identify which cells were from the sampled individual, and which were from its twin. In doing so, they demonstrated that sibling chimerism is limited to cells from the two lineages (myeloid and lymphoid) that hematopoietic stem cells give rise to. Across the organs studied, all the other cell types examined originated from the twin being sampled (Figure 1).

**Figure 1.**
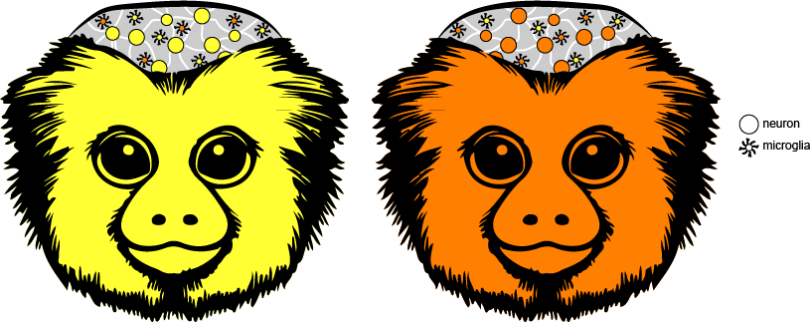
Investigating sibling chimerism in marmosets. Marmosets almost always produce non-identical twins (also known as fraternal or dizygotic twins). However, these siblings are more alike than expected because they share a circulatory system in the womb, which allows them to exchange hematopoietic stem cells. As a result, the tissues and organs of the adult twins can contain cells carrying the genetic information of their sibling (orange cells in the yellow individual, and vice versa). Single-nucleus RNA sequencing approaches allowed del Rosario et al. to investigate which tissues contained cells from both twins, and in which proportion. This showed that only cells derived from hematopoietic stem cells display such ‘sibling chimerism’. In the brain, for example, the team found evidence for this phenomenon primarily in just one type of cell – immune cells known as microglia – with other cell types (such as neurons) being solely from the individual sampled.

The team then turned its attention to microglia, the primary immune cells of the brain, using a single-nucleus RNA-sequencing dataset of more than 2.2 million cells from various brain regions of 11 marmosets (Krienen et al., 2023). Consistent with the findings for non-brain tissues, this analysis first revealed that the proportion of sibling-derived microglia varies between the brain areas of an individual. del Rosario et al. suggest two possible explanations for this finding: (1) Such variations may be due to cell migration and proliferation taking place in a random manner, with the level of sibling-derived cells reflecting which cells arrived first and multiplied the most in a tissue. (2) Alternatively, cells from the twin may be recruited and proliferate differently across brain tissues and even local environments due to their genetic background.

To then test if genetic variations between cells could indeed impact their expression patterns, del Rosario et al. directly compared how microglia in the same brain region expressed their genes depending on whether they were from the sampled animal or its twin. When assessing if cells facing the same constraints respond differently because of variations in their genomes, this is as environmentally controlled a study can get in a living animal – nature’s very own common garden experiment, where scientists examine how populations of different genetic origins fare when facing the same conditions. The results show that the local environment, such as which brain region the cells were in, had a much larger impact in shaping gene expression than the genetic background. This does not indicate that genetic variation is unimportant, but it does highlight how cells – no matter who they come from – are very good at doing their job when they find themselves in the right place at the right time.

All told, this thoughtful and thorough study accomplishes two important goals. First, it all but closes a previously open question on the extent and cell origins of sibling chimerism. Second, it sets the stage for using this unique model system to examine, in a natural context, how genetic variation in microglia may impact brain development, function, and disease.
